# The impact of inflammation and acute phase activation in cancer cachexia

**DOI:** 10.3389/fimmu.2023.1207746

**Published:** 2023-10-31

**Authors:** Tyler P. Robinson, Tewfik Hamidi, Brittany Counts, Denis C. Guttridge, Michael C. Ostrowski, Teresa A. Zimmers, Leonidas G. Koniaris

**Affiliations:** ^1^ Department of Surgery, Indiana University School of Medicine, Indianapolis, IN, United States; ^2^ Department of Surgery, Oregon Health Sciences University, Portland, OR, United States; ^3^ Hollings Cancer Center, Medical University of South Carolina, Charleston, SC, United States

**Keywords:** cachexia, cancer, acute phase response, acute phase reactant, positive acute phase reactant, negative acute phase reactant, chronic disease

## Abstract

The development of cachexia in the setting of cancer or other chronic diseases is a significant detriment for patients. Cachexia is associated with a decreased ability to tolerate therapies, reduction in ambulation, reduced quality of life, and increased mortality. Cachexia appears intricately linked to the activation of the acute phase response and is a drain on metabolic resources. Work has begun to focus on the important inflammatory factors associated with the acute phase response and their role in the immune activation of cachexia. Furthermore, data supporting the liver, lung, skeletal muscle, and tumor as all playing a role in activation of the acute phase are emerging. Although the acute phase is increasingly being recognized as being involved in cachexia, work in understanding underlying mechanisms of cachexia associated with the acute phase response remains an active area of investigation and still lack a holistic understanding and a clear causal link. Studies to date are largely correlative in nature, nonetheless suggesting the possibility for a role for various acute phase reactants. Herein, we examine the current literature regarding the acute phase response proteins, the evidence these proteins play in the promotion and exacerbation of cachexia, and current evidence of a therapeutic potential for patients.

## Graphical Abstract

The acute phase response in cachexia.

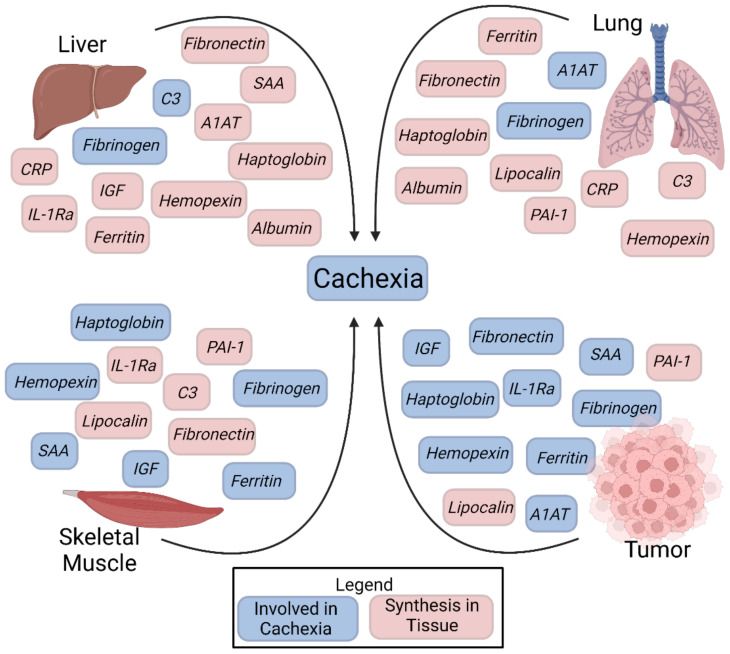

## Introduction

Cachexia is a devastating process that accompanies chronic inflammatory illnesses such as cancer, sepsis, chronic obstructive pulmonary disease (COPD), chronic heart failure (CHF), rheumatoid arthritis (RA), and chronic kidney disease (CKD.) Clinically, cachexia is diagnosed as a weight loss greater than 5%, weight loss greater than 2% in individuals with BMI < 20 kg/m^2^, or sarcopenia (1). Cachexia is classically described in cancer and is prevalent in up to 80% of cancer patients, however cachexia also exacerbates chronic illness such as COPD with prevalence as high as 15% of patients (2). Predictive scores have been developed which utilize serum inflammatory markers, nutritional parameters, weight loss, and muscular evaluation to identify those with cachexia and those who are at risk (3–5).

Cachexia is driven by an inflammatory response involving cross-talk between many organs throughout the body. The acute phase response (APR) involves changes in plasma protein concentrations as well as behavioral, psychological, biochemical, and nutritional changes in the organism. A plasma protein whose concentration increases or decreases by at least 25% in response to inflammation is defined as an acute phase protein (6). These proteins are created in response to inflammation as a result of tissue insult and function to restore tissue homeostasis and repair, including regulating cell proliferation, scar formation, and immune defensive functions. Acute phase protein synthesis is initiated by cytokine signaling, mainly through interleukin-6 (IL-6), IL-1, TNF Alpha (TNFa), and Interferon Gamma (IFNg). Previous studies have extensively described the acute phase proteins (APP) produced by the liver. APP it is divided into positive acute phase proteins and negative acute phase proteins. Positive APPs levels increase during the APR whereas negative APPs levels decrease (6–8). Overall, APP concentrations are affected during cancer (**Table 1**) as well as many other inflammatory states. To date, less is known how these factors regulate metabolism, muscle and fat homeostasis.

**Table 1:** Acute phase reactant proteins produced by the liver, lung, skeletal muscle, and tumor microenvironment.

Acute Phase Protein Name	Liver Synthesis	Lung Synthesis	Skeletal Muscle Synthesis	Tumor and Tumor Microenvironment Synthesis	Expression change correlative to cachexia	Demonstrated role In Cachexia
**Albumin**	*G,P*	*R-G (* 9) *M-P* (10)			*H-G (* 11) *H-P* (3, 4, 12)	*No*
**Alpha 1 Acid Glycoprotein**	*G,P*	*M-G (* 13) *H-P* (14)		*H-P (* 14)		*No*
**Alpha 1 Antichymotrypsin**	*G,P*		*H-P (* 15)			*No*
**Alpha 1 Antitrypsin**	*G,P*	*H,M-P (* 16)		*H-P (* 17)	*H-G (* 18)	*No*
**Alpha 1 Protease Inhibitor**	*G,P*	*H-G (* 19, 20) *H-P* (19, 20)		*H-P (* 21)		*No*
**Alpha 2 HS Glycoprotein**	*G,P*		*GP-G (* 22)	*H-G (* 23) *H-P* (23)		*No*
**Alpha Fetal Protein**				*H-P (* 24–26)		*No*
**Angiotensinogen**	*G,P*	*R-G (* 27, 28) *R-P (* 29)	*M-G (* 30) *M-P* (31)	*H-P (* 29, 32)		*No*
**C3**	*G,P*	*H,M-G (* 33) *H-P* (34)	*H-G (* 35) *H-P* (35)	*H,M-G (* 36, 37) *H-G* (38) *H,M-P* (36) *H-P* (38) *M-P* (39)	*M-G (* 40) *M-P* (40) *H-P* (41)	*No*
**CD14**	*G,P*	*M-G (* 42, 43) *H-G* (44) *M-P* (42)	*H-G (* 44)	*H-G (* 45) *H,M-P* (46)		*No*
**Ceruloplasmin**	*G,P*	*M-G (* 47) *R-G* (48) *R-P* (48)		*M-G (* 47) *H-G* (49) *M-P* (47) *H-P* (49)		*No*
**CRP**	*G,P*	*H-G (* 50, 51) *H-P* (50, 51)			*H-P (* 52, 53)	*No*
**Factor XII**	*G,P*	*H-G (* 54) *H-P* (54)				*No*
**Ferritin**	*G,P*	*H-G (* 55, 56) *H-P* (55–57)	*R-G (* 58) *M-G* (59) *M-P* (59)	*H-P (* 60, 61)	*M-G (* 62, 63) *H,M-P* (64) *H-P* (65)	*No*
**Fibrinogen**	*G,P*	*F-G (* 66) *H-G* (67) *F,H-P* (66) *H-P* (67)	*M-P (* 68)	*H-G (* 69, 70) *M-G* (71) *H-P* (69, 70) *M-P* (71)	*M-G (* 68) *M-P* (68)	*Yes (* 68)
**Fibronectin**	*G,P*	*H-G (* 72, 73) *H-P* (72, 73)	*R-P (* 74) *M-P* (75)	*H-P (* 76)	*M-P (* 77) *H-P* (78)	*No*
**Granulocyte Colony Stimulating Factor**	*G,P*	*H-G (* 79) *H-P* (80)	*M-G (* 81, 82)	*H-G (* 83) *M-G* (84) *D-G* (85) *H-P* (86, 87)		*No*
**Haptoglobin**	*G,P*	*H-G (* 88) *H-P* (88)	*M-G (* 40) *M-P* (40, 89)	*H-G (* 26, 88, 90) *H-P* (26, 88, 90)	*M-G (* 40) *M-P* (40, 68, 91) *H-P* (92)	*No*
**Hemopexin**	*G,P*	*R-G (* 93) *R-P* (93) *M-P* (94)	*M-P (* 95)	*H-P (* 96)	*M-G (* 68) *M-P* (40, 97)	*No*
**Insulin Like Growth Factor**	*G,P*		*H-G (* 98, 99) *H-P* (98, 99)	*H-P (* 100, 101) *M-P* (102)	*M-G (* 103) *M-P* (103)	*No*
**Inter Alpha Trypsin Inhibitors**	*G,P*	*H-P (* 104)				*No*
**Interleukin 1 Receptor Antagonist**	*G,P*	*H-G (* 105) *H-P* (105, 106)	*H-P (* 107)	*M-G (* 108) *M-P* (108, 109)	*M-P (* 110)	*No*
**Lipocalin**	*G,P*	*H-G (* 111) *M-G* (47)	*M-G (* 112) *M-P* (112)	*H-G (* 111, 113, 114) *M-G* (115) *H-P* (111, 114) *M-P* (115)	*M-G (* 68) *M-P* (116)	*Yes (* 116)
**Lipopolysaccharide Binding Protein**	*G,P*			*H-P (* 117, 118)		*No*
**Pancreatic Secretory Trypsin Inhibitor**	*G,P*	*H-P (* 119)				*No*
**Plasminogen**	*G,P*					*No*
**Plasminogen Activator Inhibitor 1**	*G,P*	*H-G (* 120) *M-G* (121) *H-P* (122) *M-P* (121)	*H-G (* 123) *H,R-G* (124) *H-P* (123)	*H-P (* 125)	*R-P (* 126) *M-P* (127)	*No*
**Protein S**	*G,P*		*M-P (* 128)	*H-P (* 129)		*No*
**Retinol Binding Protein 4**	*G,P*	*R-G (* 130)	*R-G (* 130)	*H-P (* 131, 132) *M-P* (133)		*No*
**Secreted Phospholipase A2**	*G,P*	*H-G (* 134, 135) *H-P* (134, 135)	*H-G (* 136) *H-P* (137)	*H-P (* 109, 138)		*No*
**Serum Amyloid A**	*G,P*	*H-G (* 139) *Rb-G* (140) *H-P* (139)	*H,M-G (* 141) *H,M-P* (141)	*H-G (* 142) *H-P* (142, 143)	*M-G (* 68) *M-P* (68)	*No*
**Thyroxine Binding Globulin**	*G,P*			*H-P (* 144)		*No*
**Tissue Plasminogen Activator**	*G,P*					*No*
**Transferrin**	*G,P*	*B,H,M-G (* 145) *R-G* (146) *R-P* (146)	*M-P (* 147) *R-P* (148)			*No*
**Transthyretin**	*G,P*		*R-G (* 130) *M-G* (149) *M-P* (150)	*H-G (* 151) *H-P* (151) *M-P* (152)		*No*
**Urokinase**			*H-G (* 123) *H-P* (123) *M-P* (153)	*H-G (* 154) *H-P* (155)		*No*
**Vitronectin**	*G,P*	*M-G (* 156)	*M-G (* 156)	*H-G (* 157, 158) *H-P* (157, 158)		*No*

The acute phase reactants that were studied in cachexia are listed on the final row. The first letter in each cell indicates the study model; H denotes human, M denotes mouse, R denotes rat, GP denotes guinea pig, F denotes ferret, D denotes dog, Rb denotes rabbit, B denotes baboon. The second letter indicates if the results found were genotypic or phenotypic. P denotes if a study mentioned protein expression found in the tissue and G denotes if a study found genetic expression in the tissue.

The APR has been shown to increase the patient’s resting energy expenditure, a feature of elevated catabolism (159). This supports the assertion APPs have a molecular role in the progression of cancer and chronic illness related cachexia. The liver is not the only organ capable of synthesizing proteins in the APR (160), and understanding the APR relationship to cachexia remains poorly defined. Therefore, we sought to review current understanding of how the APR promotes cachexia. Currently, we could identify no studies demonstrating the *in vivo* manipulation of APP activation and improvement or other effects on the cachectic response. In our review of the literature, we sought a comprehensive list of acute phase proteins. We used the well-established list of acute phase proteins described by Gabay and Kushner (6) as well as our expert judgement as the basis for our literature search and review. Through our expert consensus we have identified CD-14 and lipocalin as APP of interest and included these in our examination. Herein, we review our current understanding of the positive and negative acute phase response related proteins and their possible role in promoting cachexia. We specifically examine APP production and presence in extrahepatic tissues including lung, skeletal muscle, and tumor microenvironment as contributors towards cachexia.

## Inflammation the acute phase response and their relationship to cachexia, a central role for IL-6

Inflammation is responsible for protein production during the acute phase response which critically requires IL-6 to produce many APP (161).Cytokines are the primary signaling mechanism by which the up and down regulation of acute phase proteins occurs. Innate immune cells are activated through toll like receptors to produce IL-1 and TNF alpha. These cytokines lead to further release of IL-6 (162, 163). IL-6 stimulation of hepatocytes produces increased positive acute phase reactants such as CRP, SAA, Alpha 1 anti-chymotrypsin, fibrinogen and decreased negative acute phase reactants such as albumin, transferrin, and fibronectin (161). IL-6 also has been shown to directly cause cachexia through a cross-talk mechanism between tumor, muscle, and fat requiring IL-6 signaling (164). Inflammation is the dominant driver of cachexia as the body struggles to maintain the balance of host defense versus the negative impacts of immune defenses on the host itself (165, 166). Measurement of the inflammatory response may be done through surrogate acute phase proteins such as C Reactive Protein (CRP), which is considered the most widely accepted index of systemic inflammation (1). CRP has been predictive of degree of cachexia as well as survival in cancer patients (3, 167). CRP is one of many examples of acute phase reactant that are a byproduct of inflammation and have a relationship to cachexia. There are many examples of regulation of acute phase response signaling in cachexia (168). In the following sections we describe extra-hepatic organ, muscle, and tumor production of such acute phase reactants.

## Acute phase reactant proteins in cachexia

In this section, we document the APP described in the literature as potential mediators of cachexia. Table1 provides a comprehensive, current summary of the literature regarding APP, their tissue of origin, and current evidence for a role in inducing cachexia.

## Positive acute phase reactant proteins in cachexia

### Alpha 1 antitrypsin

Alpha 1 antitrypsin(A1AT) is synthesized in the liver and serves to inhibit neutrophil elastase in the lung (169). A1AT protein is also produced by the lung and tumor microenvironment of lung cancer where it promotes metastasis, chemotherapy resistance, and decreases survival (16, 17). Although alpha 1 antitrypsin deficiency correlates with pulmonary cachexia (18), a clear role for A1AT has not been established in cancer cachexia.

### Complement

The complement system is a critical component of innate and adaptive immunity. It consists of about 30 proteins that follow three different pathways to provide many functions such as defense against pathogens, opsonization, chemoattraction, apoptosis, and thrombosis (170). Historically, complement has been described as a product of the liver (171). Complement C4, C9, Factor B, C1 inhibitor, C4b-binding protein, and mannose binding lectin are recognized as acute phase proteins, however only factor B has been shown to participate in the tumor microenvironment (172) and none are synthesized by the lung or skeletal muscle according to the current literature. C3 is unique, in that all three pathways converge on this one common downstream protein, and because it has the most data regarding cachexia, we will discuss it further.

### C3

C3 has been shown to be synthesized by the lung, skeletal muscle, and is detected in the tumor microenvironment (33–39). The only study that demonstrated extrahepatic production of complement utilized the colon-26 carcinomas bearing mice (C26 mouse model) which develop cancer cachexia. This study showed increased skeletal muscle production of C3 (40). C3 has been shown in clinical studies to be upregulated in cachexia. Pre-operative pancreatic cancer patients who had cachexia and elevated CRP had an increase in serum C3a compared to non-cachectic pancreatic cancer patients (41). Complement, specifically C3, serves as a candidate for further research in cachexia. Currently, there are no functional studies which evaluate its role in cachexia.

### CRP

CRP is one of the most measured APP that is secreted by the liver. CRP functions in the immune response in opsonization, phagocytosis, and cytokine signaling (173, 174). CRP mRNA and protein is expressed in the lung, however not in the skeletal muscle or tumor microenvironment (50, 51). When myoblasts are exposed to high levels of CRP, cell proliferation is inhibited (175). One challenge of examining the contribution of CRP to cachexia is that it is not synthesized in murine models of cachexia. Therefore, the relationship observed between CRP and cachexia is predominantly limited to clinical studies. In clinical studies of cachexia, the acute phase response is synonymous with CRP. Pro-inflammatory cytokines induce the APR and in turn CRP (7, 159, 176, 177). Specifically, IL-6 impacts CRP as shown by a reduction in CRP serum levels in weight-losing cancer patients by blocking IL-6 (52). In patients with pancreatic cancer who underwent neoadjuvant chemoradiation therapy, CRP was increased and a value of greater than 10 kU/L was associated with reduced survival (53). Fearon et al. has also noted that CRP may offer prognostic value in cachexia (1). The Glasgow Prognostic Score utilizes CRP as a predictor of cachexia severity (3). The modified Glasgow Prognostic Score has been proposed to identify pre-cachectic patients that may benefit from multimodal therapy to benefit the onset of cachexia (178). CRP has a role in clinical management of patients with cachexia, however it will be difficult to determine an underlying mechanism without murine studies.

### Ferritin

Ferritin is an APP involved in the cellular stress response as well as iron regulation and homeostasis by storing iron and releasing it during times of cellular need (179). It is mainly produced in the liver. In chronic inflammatory states such as cancer, in addition to ferritin, cytokines upregulate hepcidin which acts to block iron absorption (180). Cytokines inhibit the proliferation of erythroid progenitor cells in bone marrow and erythropoietin production by the kidney. Ultimately all of these mechanisms lead to anemia of chronic disease, a common condition in cachexia (181) Currently, erythropoiesis-stimulating-agents such as epopoetin alpha, which has similar actions as endogenous erythropoietin, are given to treat this anemia (182). The series of interactions with ferritin, hepcidin, and iron homeostasis serve as potential targets for cachexia therapy that may alleviate the morbidity of anemia of chronic disease. The lung expresses ferritin mRNA and protein when exposed to stressed conditions. The skeletal muscle increases ferritin mRNA after denervation, it increases protein expression when exposed to adiponectin, and the tumor microenvironment displays ferritin protein accumulation (55–61). *In-vitro* models of cancer cachexia have demonstrated upregulated ferritin mRNA and protein expression in various tumor cell lines (62–64). In cachectic patients with gastric cancer, ferritin protein expression was upregulated in skeletal muscle compared to non-cachectic gastric cancer patients (65). Finally, it has been shown that by providing iron supplement in both mice and human patients with cancer cachexia, muscle function and strength improved (64). Ferritin has demonstrated evidence of involvement in cachexia both preclinically and clinically, and further studies should target the ferritin and hepcidin mechanisms of iron storage in chronic disease to increase bioavailability of iron in cachectic patients.

### Fibrinogen

The majority of fibrinogen is predominantly synthesized in the liver and is involved in coagulation where it is cleaved into fibrin to form a platelet plug. The platelet plug can also trap other cell types including erythrocytes and leukocytes in pathological situations (183). Fibrinogen is produced extrahepatically in the lung during inflammation, skeletal muscle during cachectic catabolism, and in the tumor microenvironment where it is deposited from serum as well as synthesized by tumor cells themselves (66–68, 184, 185). Fibrinogen has been studied extensively in cachexia. Initial work by O’Keefe and colleagues identified protein turnover in cachexia contributes to fibrinogen production (186). Preston and colleagues demonstrated that using labeled phenylalanine, increase in circulating amino acids from skeletal muscle breakdown were ultimately used for fibrinogen synthesis in patients with pancreatic adenocarcinoma (187). Functional studies of cancer cachexia mice treated with C26 tumors had increased serum fibrinogen, however the synthesis was assumed to be hepatic (188). Another study of C26 treated mice performed a secretome analysis on the skeletal muscle and identified fibrinogen as potentially being synthesized and secreted by the skeletal muscle. Bonetto and colleagues have shown that myotubes exposed to IL-6 induced STAT3 signaling which coincided with increased fibrinogen protein expression (68). Fibrinogen production was increased as cachexia severity worsened in the C26 mouse model. Given that fibrinogen is produced by many extrahepatic tissues and is a protein of interest in cancer cachexia, it remains a protein of great interest in future studies on the APR in cachexia.

### Fibronectin

Fibronectin is mainly synthesized by the liver and has several functions. It is involved in extracellular matrix cell adhesion, it allows for host defense through participation in the immune response, and it stabilizes clot in thrombogenesis (189). Fibronectin mRNA and protein is expressed in the lung during inflammation, the protein is found in normal and regenerating skeletal muscle, and the extracellular matrix of the tumor microenvironment influencing tumor spread and growth (72–74, 76, 190, 191). Fibronectin has been examined in cachexia models. Transforming growth factor-beta (TGF-b) null mice experienced leukocyte infiltration in the heart, lungs, pancreas, stomach, colon, and salivary glands that ultimately lead to cachexia. This immune response was reduced by daily administration of fibronectin, which may have impeded the ability of leukocytes to invade target tissues (77). Fibronectin was found to be deposited in subcutaneous adipose tissue from gastrointestinal cancer patients with cachexia. There was also increased TGF-b found in these samples suggesting that TGF-b signaling may have resulted in fibronectin deposition (78). Further investigation of the inflammatory response, fibronectin, and cachexia may elucidate an underlying mechanism. The current evidence does not offer a clear mechanism at this time.

### Haptoglobin

Haptoglobin is a protein produced by the liver that scavenges and binds to hemoglobin, the oxygen carrying component of red blood cells, after destruction of red blood cells. Haptoglobin may also play a role in host defense during the inflammatory response (192). Haptoglobin is synthesized by the lung during inflammation, skeletal muscle during cachexia, and tumor microenvironment where it causes cellular changes, which promote metastasis (40, 88, 90). Haptoglobin protein levels are increased in a ciliary neurotrophic factor induced cachexia rat model and cachexia associated with congestive heart failure in human patients (91, 92). Bonetto and colleagues have also shown an increased serum haptoglobin in the C26 cancer cachexia mouse model (68), in addition to several other acute phase proteins. Massart and colleagues demonstrated similar findings (40). Analysis of the contribution of lung and tumor microenvironment derived haptoglobin in cachexia serves as an area for future research.

### Hemopexin

Similar to haptoglobin, hemopexin is mainly synthesized in the liver and binds to heme after hemolysis to prevent toxicity from generation of reactive oxidative species (193). Hemopexin is expressed in the lung during inflammation and infection, skeletal muscle during atrophy, and it is present in the tumor microenvironment where it is associated with metastatic disease (93–96). There are several studies of cancer cachexia models that demonstrate a relationship to hemopexin. Proteomic profiling of C26 cancer cachexia mice gastrocnemius show an increased expression of a hemopexin precursor (97). This is consistent with other C26 data that demonstrates a 17-fold increase in hemopexin gene expression in skeletal muscle as well as almost 9000 times more hemopexin protein found in C26 skeletal muscle compared to controls (40, 68). Although these studies are associative a clear mechanistic link has yet to be demonstrated.

### Interleukin 1 receptor antagonist

As the name suggests, Interleukin 1 Receptor Antagonist (IL-1Ra) is an acute phase protein produced by the liver, inhibits the IL-1 receptor and thereby acts as an anti-inflammatory mechanism (194, 195). In addition to synthesis by the liver, alveolar macrophages in the lung synthesize IL-1Ra, the protein has been detected in the skeletal muscle of patients with the muscular inflammatory diseases, dermatomyositis and polymyositis. High IL-1Ra levels were detected within the tumor microenvironment after immunotherapy and its anti-inflammatory properties may promote cancer growth (105–109). IL1-Ra has a role in cachexia however it may not directly affect tumor growth. IL1-Ra injected intratumorally in a C26 cancer cachexia model reduced IL-1 signaling within the tumor and increased mouse lean mass and fat mass, at least partially reversing the wasting effects of cachexia (110). IL-1Ra requires further evaluation as an APP in cachexia, but it has shown promise that an underlying mechanism may exist.

### Lipocalin 2

Lipocalin 2 is also known as neutrophil gelatinase-associated lipocalin and is a member of the lipocalin superfamily. Lipocalin 2 is mainly produced by neutrophils and is involved in the inflammatory response as well as metabolism (116). Lipocalin 2 also occurs in the lung, which produces lipocalin mRNA. Skeletal muscle produces lipocalin mRNA and protein in ob/ob mice, and the tumor microenvironment has increased lipocalin mRNA and protein (47, 111–115). Lipocalin mRNA has been upregulated in the quadriceps of mouse model of cachexia (68). Lipocalin has been further studied in a mouse model of cachexia and was shown to control cachexia-anorexia by inhibiting appetite and exacerbating the cachexia phenotype. Infusion of an antagonist for the lipocalin receptor reversed cachexia. Interestingly, this study also correlated lipocalin levels in patients with pancreatic cancer and found increased mortality in the setting of lipocalin upregulation (116). This study implicates lipocalin as a regulator of cachexia metabolism, and future studies should attempt to evaluate lipocalin antagonists in human patients.

### Plasminogen activator inhibitor type 1

Plasminogen Activator Inhibitor Type 1 (PAI-1) is a pro-coagulation protein involved in the coagulation cascade. Tissue plasminogen activator(tPA) and urokinase plasminogen activator (u-PA) help transform plasminogen into plasmin, which dissolves fibrin clot. PAI-1 serves as an inhibitor of both tPA and u-PA (196). PAI-1 is synthesized by the liver as well as the lung, skeletal muscle, and tumor microenvironment (120–123, 125, 197). PAI-1 has a relationship to cachexia as well. PAI-1 has been shown to be elevated in the myocardium of Walker-256 carcinoma bearing cachectic mice (126). A study of radiation therapy applied to U87MG cells, a glioblastoma cell line, caused these cells to secrete PAI-1 exosomes. When C2C12 myoblasts were treated with PAI-1 exosomes they experienced muscle wasting (127). This preliminary data regarding PAI-1 in cachexia is unable to demonstrate a clear mechanism, and this remains an area of focus for research of the APR in cachexia.

### Serum Amyloid A

Serum Amyloid A(SAA) is synthesized by the liver during the APR and has many functions including both pro and anti-inflammatory actions critical for host survival (198). Increased SAA mRNA and protein are found in the lung and skeletal muscle during a state of inflammation, as well as the tumor microenvironment which may lead to worse tumor-free survival in patients (139–143). C26 cancer cachexia mice show elevated levels of both SAA mRNA and protein in the skeletal muscle (40, 68). SAA lacks a mechanistic connection to cachexia and more work should be done to evaluate SAA in cachexia.

## Negative acute phase reactant proteins in cachexia

### Albumin

Albumin is made primarily by the liver and regulates intravascular oncotic pressure and transports ligands throughout the body (199). Albumin mRNA and protein are expressed in the lung and skeletal muscle (9, 10, 200). Clinically, albumin has been studied as a biomarker to predict cachexia using scores such as the Glasgow Prognostic Score (3, 4, 12). Furthermore, there are several lines of preclinical evidence, as well. Decreased albumin in cancer cachexia is correlated to increased mortality (201). Intramuscular injections of TNFα, induced cachexia and coincided with decreased hepatic albumin synthesis (11). Of note, the decrease in albumin preceded the development of cachexia. Albumin is currently viewed as a marker of cachexia, and there is a lack of data that may explain its role in contributing to the process of cachexia.

### Insulin like growth factor 1

Insulin Like Growth Factor 1 (IGF1) is a protein that is widely produced but mainly synthesized in the liver. IGF1 is involved in growth and anabolism as well as cell homeostasis and the bioavailability of sex hormones (202). IGF expression is present in skeletal muscle during growth, development and damage repair. In the tumor microenvironment, IGF protein may lead to tumor progression (98–102). Cisplatin is a commonly used chemotherapy and is sufficient to induce muscle wasting. IGF administered in conjunction with Cisplatin prevented wasting of myotubes and mouse skeletal muscle suggesting a mechanism to prevent chemotherapy induced muscle wasting (103). In congestive heart failure-associated cachexia, skeletal muscle displays reduced IGF and reactivation of IGF signaling pathways prevented skeletal muscle proteolysis (203). IGF requires more functional data *in vitro* and *in vivo* as well as human studies specifically evaluating IGF in cancer cachexia.

## Discussion

The APR is highly active in response to inflammatory stimuli and a critical part of the innate immune system including the potentially detrimental cachexia response. APR proteins are traditionally thought to be produced mainly by the liver. However, increasing evidence demonstrates that the lung, skeletal muscle, and tumor microenvironment express large quantities of APPs at the mRNA and protein level. Furthermore, it has been shown that cachexia provides the substrates that fuel synthesis of APPs. As an example, Preston and colleagues have demonstrated amino acids from muscle produced fibrinogen is increased in cachectic patients (187). Various studies described above have associated the APR proteins with cachexia. Albumin and CRP concentration have been predictive of patient outcomes and serve as examples of APP that have clinical applicability to cachexia. Only fibrinogen and lipocalin have had a sufficiently demonstrated role in cachexia. Fibrinogen has both increased mRNA and protein expression that is synthesized by the liver and skeletal muscle through IL-6/STAT3. Fibrinogen concentration increases as severity of cachexia increases and a mechanism of STAT3 increasing fibrinogen synthesis through proteolysis resulting in cachexia has been proposed. Lipocalin offers another plausible APP-induced cachexia mechanism as lipocalin crosses the blood-brain barrier and alters feeding behavior resulting in cachexia. However, there is a clear paucity of functional experimental data surrounding the APR in cachexia and further research is needed to elucidate this relationship. Murine models are critical in cachexia research, especially in the vital role of testing treatment efficacy and safety prior to human exposure (204). These results must be interpreted with caution, as murine models may be limited in their ability to replicate the human phenotypes of cancer and their complex microenvironment. Such models are injected with tumor cell lines as opposed to spontaneous growth, develop cachexia within weeks, and grow tumors that may be proportionately larger then human tumors (205). The acute phase response serves as a candidate for a potential molecular target that may help improve patient prognosis from cancer cachexia. Expression of many APR change and correlate to cachexia, but a mechanistic link has remained elusive. Investigating the molecular mechanisms by which the APPs are involved in cachexia has the potential to identify new targets and open new strategies to help the patients overcome cancer-associated cachexia. Untangling the relationship between the APR and cachexia still requires further investigation as it remains unclear if the APR is a mediator of cachexia.

## Author contributions

TR: Primary research, draft manuscript, editing. BC: Additional research and content, editing of manuscript. TH: Additional research and content, editing of manuscript. DG: Additional research and content, editing of manuscript. MO: Additional research and content, editing of manuscript. TZ: Manuscript idea, additional research and content, editing of manuscript. LK: Manuscript idea, additional research and content, editing of manuscript. All authors contributed to the article and approved the submitted version.
